# Uterine metabolic disorder induced by silica nanoparticles: biodistribution and bioactivity revealed by labeling with FITC

**DOI:** 10.1186/s12951-021-00810-x

**Published:** 2021-02-28

**Authors:** Shuyin Duan, Meihua Zhang, Junxia Li, Jiaqi Tian, Haoyu Yin, Xietong Wang, Lin Zhang

**Affiliations:** 1 Key Laboratory of Birth Regulation and Control Technology of National Health Commission of China, Maternal and Child Health Care Hospital of Shandong Province, Shandong University, 250001 Jinan, China; 2 School of Public Health, Zhengzhou University, 450001 Zhengzhou, China; 3 School of Public Health, Weifang Medical University, 261053 Weifang, China; 4 Department of Obstetrics and Gynecology, Shandong Provincial Hospital, 250001 Jinan, China

**Keywords:** Silica nanoparticle, Biodistribution, Uterine inflammation, Unsaturated fatty acids, Trophoblast

## Abstract

Extensive application of nanomaterials has dramatically increased the risk of silica nanoparticle (SiNP, SiO_2_) exposure, yet their biological effect on reproduction has not been fully elucidated. By tracking the uterine biodistribution of SiNP in pregnant mice, this study was conducted to evaluate the biological effect of SiNP on reproduction. First, SiNP was conjugated with FITC, and then the FITC-SiNP was administrated to trophoblast (100 µg/mL, 24 h) in vitro and pregnant mice (0.25 mg/mouse, 2–24 h) in vivo. It was found that the FITC-SiNP was internalized by trophoblast and deposited in the uterus. The internalization of SiNP caused trophoblast dysfunction and apoptosis, while SiNP accumulation in the uterus induced diffuse inflammatory infiltration. The genome-wide alteration of gene expression was studied by high throughput sequencing analysis, where 75 genes were found to be dysregulated after SiNP exposure, among which ACOT2, SCD1, and CPT1A were demonstrated to regulate the biosynthesis of unsaturated fatty acids. Moreover, the suppression of unsaturated fatty acids caused mitochondrial overload of long-chain fatty acyl-CoA (LACoA), which further induced both trophoblast apoptosis and endometrial inflammation. In conclusion, the successful conjugation of FITC onto SiNP facilitated the tracking of SiNP in vitro and in vivo, while exposure to FITC-SiNP induced uterine metabolic disorder, which was regulated by the ACOT/CPT1A/SCD1 axis through the biosynthesis of unsaturated fatty acids signaling pathway. 
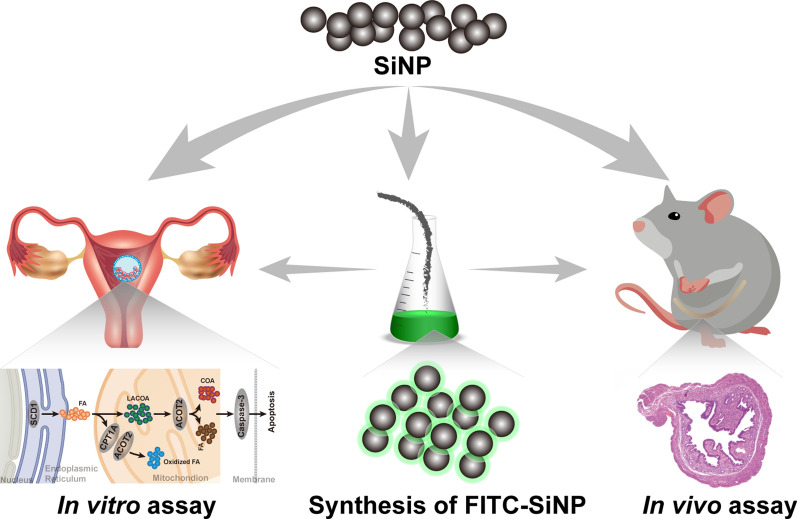

## Introduction

With the rapid development of nanotechnology, a large number of nanomaterials are produced, which immensely increases the risk of nano-dust exposure through either manufacturing or daily life routes [1]. Generally, nanomaterials are characterized by smaller than 100 nm in any dimension of their size and easy to be modified, which are widely used in areas of industrial, medical, and military applications [2]. However, the theatrical release of nanoparticles calls into question on both public health and the ecological environment. It is reported that the commercialized silver (Ag) nanoparticles are harmful to bacteria in soil [3], and phagocytosis of carbon nanotubes (CNTs) leads to accumulation of reactive oxygen species (ROS) and induces macrophage apoptosis [4]. In particular, silica nanoparticle (SiNP, SiO_2_) exposure has been proven to be associated with various physical disorders, including chronic obstructive pulmonary disease, cardiovascular diseases, infertility, and even adverse pregnancy outcomes [5–7].

As one of the most widely used nanomaterials, SiNP is defined with a diameter between 5 and 100 nm and a surface area in the range of 25–50 m^2^/g [8]. To date, SiNP is explicitly applied in gas sensors, gene delivery, biomedical therapy, and additives to cosmetics and foods, and these applications significantly increase their exposure opportunities [9], among which inhalation and dermal contact were the most common routes, especially under specific conditions such as coal mining, cement production, and welding operations [10]. Notably, the crucial role of the gastrointestinal tract in absorbing SiNP is attracting more and more attention as nanoparticles are increasingly applied in food [11], based on which it is estimated that the daily intake of SiNP ranges from 9.3 to 50.4 µg/mL [12]. Moreover, SiNP has been becoming the dominant component of atmospheric particulates, and it increases in areas with severe air pollution. Particularly, the concentration of atmospheric SiNP is closely associated with the prevalence of pulmonary diseases [13, 14].

As evidenced by previous studies, SiNP has been found to penetrate physiological barriers such as gas-blood and maternal-fetal and travel with blood flow, and this constitutes the prerequisite for assessing the impact of SiNP on reproduction [15, 16]. The pregnant uterus is rich in capillaries and has active blood flow, and it provides substantial advantages for SiNP deposition. However, uterine accumulation of SiNP is demonstrated to damage embryo implantation and fetal development [17]. Mechanistically, SiNP exposure dysregulates the related biological processes, most commonly resulting in robust oxidative stress, apoptosis, DNA damage, and inflammation. Recent studies show that the testis-deposited SiNP induces abnormal mitosis through disrupting mitochondrial metabolism [18]. To some extent, exposure to TiO_2_, ZnO, and Ag nanoparticles is also associated with infertility and a set of adverse pregnancy outcome events [19, 20]. However, based on current findings, it is still challenging to reveal the regulatory mechanism of SiNP-induced reproductive disorder.

According to guidelines put forward by the International Institute of Life Sciences (ILSIRF), this study was designed and conducted to investigate the impact of SiNP exposure on reproduction, where SiNP was first conjugated with fluorescein isothiocyanate (FITC), and then the FITC-SiNP was applied to track SiNP in vitro and in vivo. The high throughput sequencing analysis and laboratory experiments further confirmed the pathogenesis of SiNP-induced reproductive inflammation. Data obtained in this study would contribute to understanding the biological effect of SiNP better as well as developing preventive and control measures for atmospheric SiNP.

## Results

### Chemical conjugation of FITC-SiNP and effect of SiNP internalization on trophoblast in vitro

**Fig. 1:**
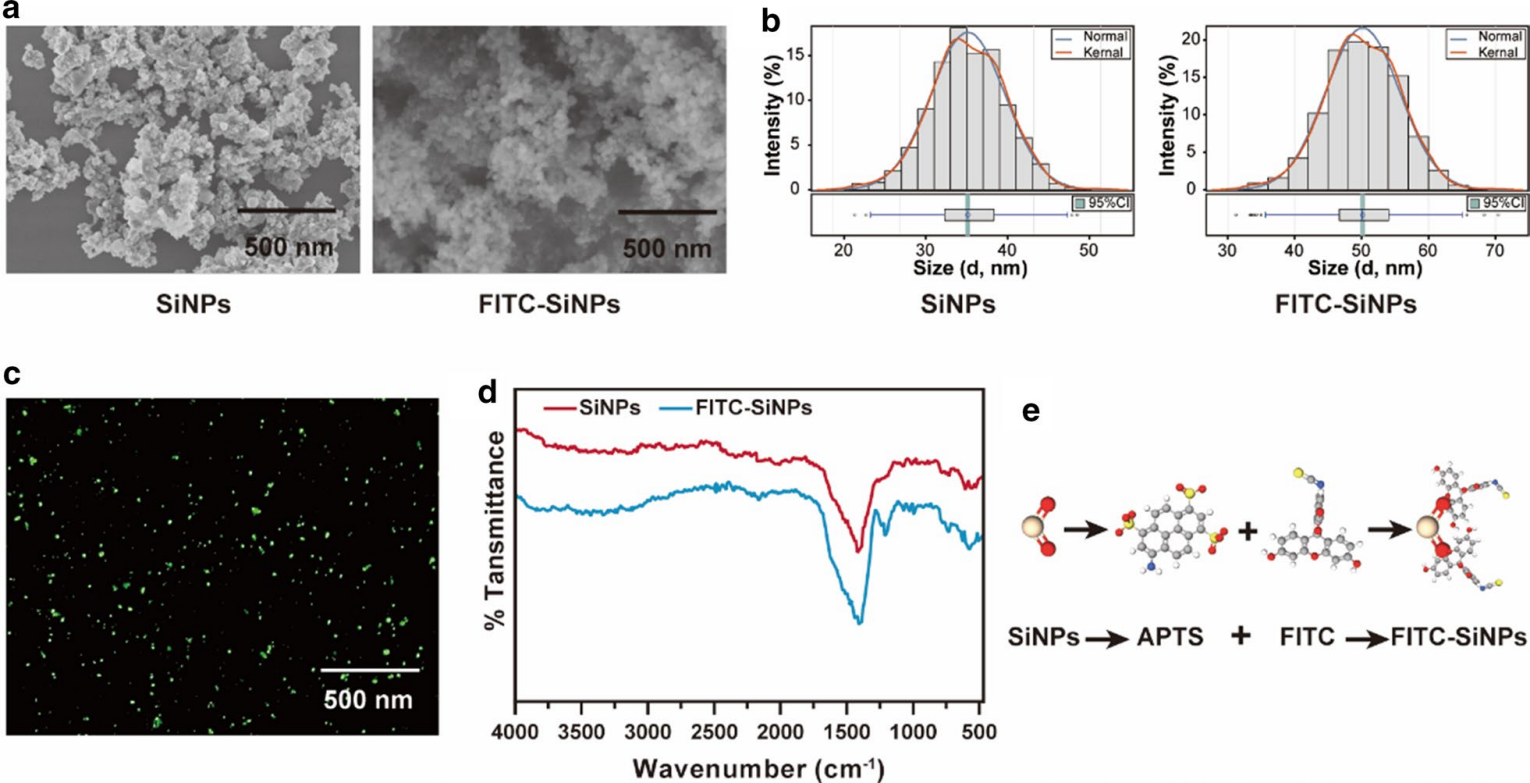
Characterization of SiNP and FITC-SiNP. **a** Images captured by scanning electron microscopy showing the morphological characteristics of SiNP and FITC-SiNP. **b** Dynamic light scattering analysis of SiNP and FITC-SiNP. **c** FITC-SiNP visualized by fluorescence microscopy. **d** Fourier transformed infrared spectra showing the transmittance curves of SiNP and FITC-SiNP. **e** Schematic illustration of the chemical synthesis of FITC-SiNP

To track SiNPs in vitro, we conjugated the FITC onto SiNP to obtain FITC-SiNP. As shown in Fig. 1a, the SiNP and FITC-SiNP were observed under a scanning electron microscopy (SEM). It was found that both SiNP and FITC-SiNP were dispersed over the observation field and were highly identical in size and shape. The dynamic light scattering (DLS) analysis was conducted to study the hydrodynamic radius of SiNP and FITC-SiNP (Fig. 1b), where the radius of SiNP slightly increased after FITC conjugation (35.71 ± 4.86 nm vs. 50.16 ± 5.55 nm). For nanoparticles suspended in cell medium, the particle size of FITC-SiNP was similarly increased, in combination with the corresponding Zeta potential and polydispersity index that were listed in Additional file 1: Table S1, all of which indicated an excellent monodispersity and stability of both nanoparticles in different solutions. Moreover, the FITC-SiNP was visualized by immunofluorescence microscopy and showed in Fig. 1c, and the qualitative and quantitative analysis of SiNP and FITC-SiNP was conducted using Fourier transform infrared spectrometer (FTIR) and X-ray diffraction (XRD). For FTIR, two strong absorption peaks at 1395 and 586 cm^− 1^ were identified based on previous studies, and they were probably generated by the asymmetric stretching vibration and bending vibration of Si-O-Si and were used to indicate SiNP and FITC-SiNP. Notably, a specific absorption peak at 1206 cm^− 1^ was also determined in FITC-SiNP, which might be generated by Si-OH stretching vibration (Fig. 1d). Also, XDR showed a relatively strong reflecting peak in the 2*θ* region of 10–30 °, which reflcted the structural property of SiNP (Additional file 1:Figure S1).

**Fig. 2:**
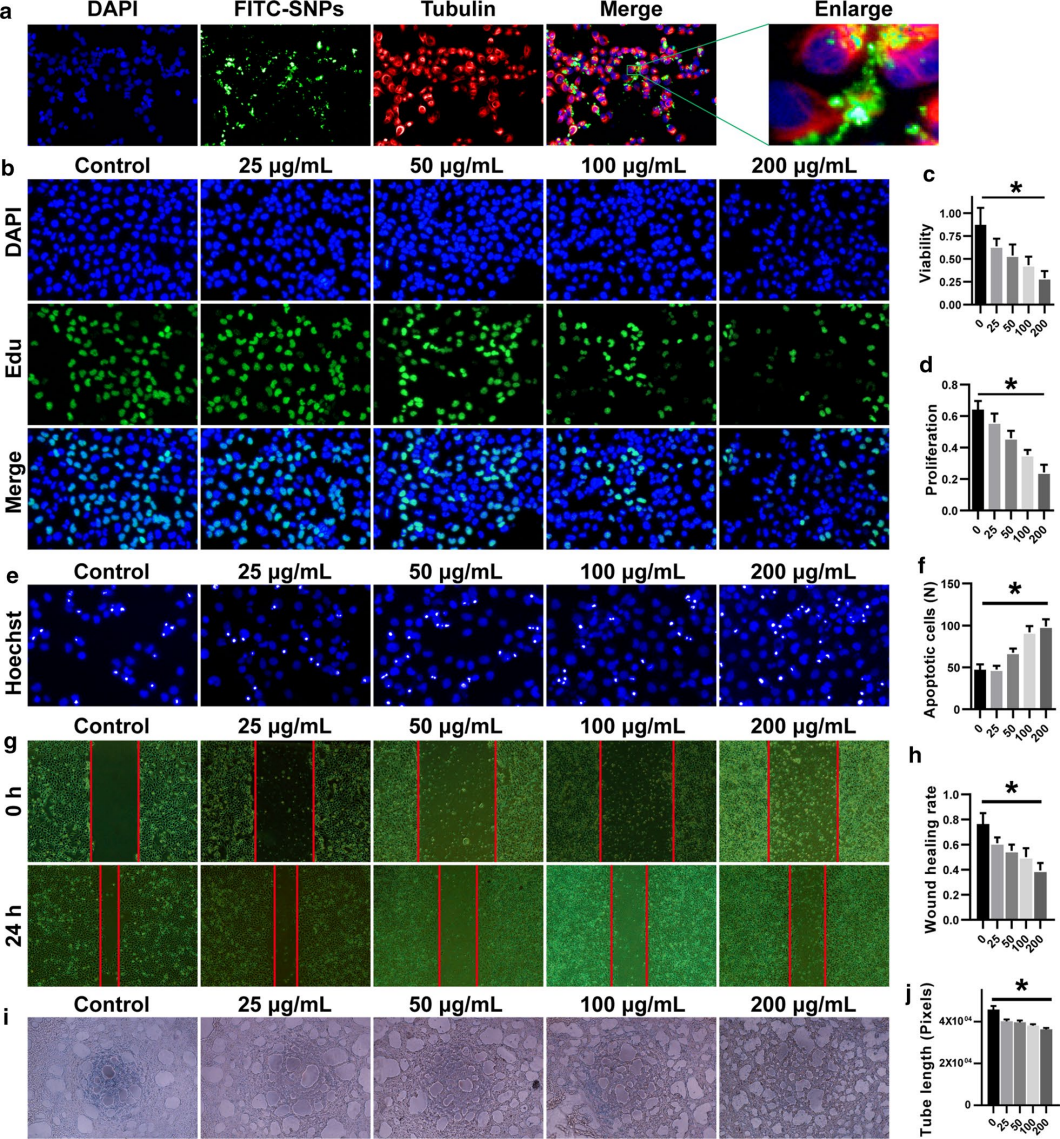
Impact of SiNP internalization on the viability and biological function of the HTR8 cells. **a** Images showing the biodistribution of FITC-SiNP internalized by the HTR8 cells (×10 magnification). The nuclei were stained with Hoechst in blue, SiNP was conjugated with FITC in green, and the cytoskeleton was stained with Tubulin in red. **b** Images showing the suppressed proliferation of the HTR8 cells, where the HTR8 cells were treated with SiNP without FITC conjugation, the nuclei were stained with DAPI in blue, the HTR8 cells in the proliferation stage were stained with EdU in green (×10 magnification). **c** CCK8 assay showing the attenuated cell viability of the SiNP-exposed HTR8 cells. **d** Quantitative analysis of the proliferation of the HTR8 cells. **e** Confocal laser scanning images showing the apoptotic HTR8 cells, where the apoptotic cells were labeled with white dots (×10 magnification). **f** Quantitative analysis of apoptotic HTR8 cells. **g** Images showing the wounds scratched in the HTR8 cells. The attenuated wound healing ability was represented by the reduced area of the wound (×10 magnification). **h** Quantitative analysis of wound healing ability of the HTR8 cells. **i** Optical microscopy images showing tubes formed by the HTR8 cells (×4 magnification). **j** Quantitative analysis of the total tube length formed by the HTR8 cells. The differences were compared using one-way ANOVA followed by SNK post-hoc test. *P < 0.05

Since trophoblast composes the outermost epithelial layer of blastocyst, it is widely used in studying the biological effect of xenobiotics on reproduction and fetal development [21]. As shown in Fig. 2a, the FITC-SiNP was found in the inner part of the HTR8 cells after exposure for 2 h. However, exposure to SiNP significantly reduced the viability of the HTR8 cells (Fig. 2c), and the alteration of cell viability was further investigated using 5-ethynyl-2′-deoxyuridine (5′-EdU) proliferation assay and Hoechst assay. Consistently, the number of EdU-stained HTR8 cells decreased in a dose dependent manner (Fig. 2b, d). In contrast, the number of Hoechst-stained HTR8 cells increased (Fig. 2e, f). Moreover, the effects of SiNP exposure on the biological function of trophoblast were studied from two aspects of cell migration and tube formation. As shown in Fig. 2 g-j, the invasion and migration of the HTR8 cells decreased after SiNP exposure, so as the total tube length formed by the HTR8 cells. These data suggest that FITC-SiNP is successfully generated and engulfed by the HTR8 cells, and the internalization of SiNP suppresses cell proliferation and triggers cell apoptosis, which adversely affects the viability and biological function of the HTR8 cells.

### Genome-wide alteration of gene expression in trophoblast exposed to SiNP

**Fig. 3:**
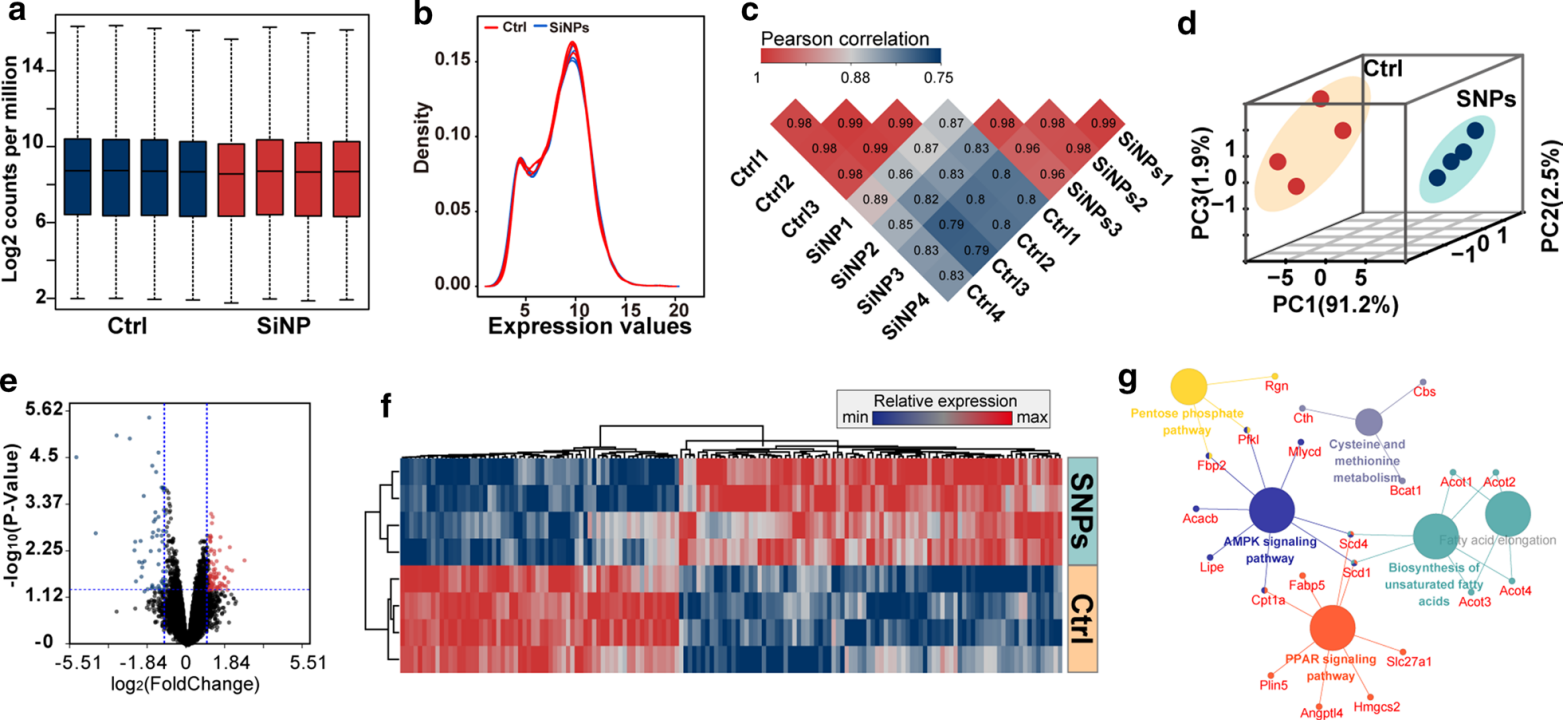
High throughput sequencing and bioinformatics analysis of HTR8 cells with different interventions. **a** Boxplot showing the gene expression of HTR8 cells. Four samples of the HTR8 cells were exposed to SiNP at 100 µg/mL for 24 hours, and the other four samples were exposed to SiNP-free cell medium and were used as controls. Ctrl, control group; SiNPs, SiNP exposure group. **b** Density plot showing the gene expression of each sample. **c** Pearson correlation analysis between HTR8 cells with different interventions. The coefficients between different samples in the same group (red) were much higher than that in different groups (blue). **d** Principal component analysis of HTR8 cells with different interventions. All samples were clustered into two groups in accordance with their actual treatments. **e** Volcano plot showing the expression of genes in the HTR8 cells. Green, downregulation; red, upregulation; black, not significant. **f** Heatmap showing the dysregulated genes in SiNP-exposed HTR8 cells. The clustering analysis was conduced among different samples or dysregulated genes. Blue, downregulation; red, upregulation. **g** Topological network of the functional annotation and enrichment analysis based on the dysregulated genes. Different functional modules were labeled with different colors

To unveil the molecular mechanism of SiNP-induced trophoblast dysfunction, we profiled the expression of genes at the genome-wide level and conducted bioinformatics analysis. A total of eight samples of HTR8 cells were recruited, where four samples were exposed to SiNP at 100 µg/mL, and the other four samples in the control group were treated with SiNP-free medium. As shown in Fig. 3a, a total of 16,448 genes were identified, and a density plot showing the expression of these genes was drawn in Fig. 3b. In addition, the correlation coefficients between samples in the same group were much higher than those between different groups (Fig. 3c). Two components were successfully concluded from principal component analysis (PCA), and they were rigorously consistent with the actual grouping method (Fig. 3d), suggesting that SiNP significantly changed gene expression of the HTR8 cells, which could be used to distinguish normal cells from the SiNP-exposed ones.

Utilizing bioinformatics analysis, 75 genes were identified to be aberrantly expressed, of which 32 genes were upregulated, and 43 genes were downregulated (Fig. 3e, f, Additional file 1: Table S2). The functional enrichment analysis was conducted to predict the potential regulatory mechanism of SiNP-induced disorders, where a total of 6 signaling pathways were identified, including the pentose phosphate pathway, cysteine and methionine metabolism signaling pathway, PPAR signaling pathway, AMPK signaling pathway, fatty acid elongation signaling pathway, and biosynthesis of unsaturated fatty acids signaling pathway (Fig. 3g, Additional file 1: Table S3). Using the false discovery rate (FDR) as an indicator for controlling the expected proportion of falsely rejected hypotheses [22], the potential regulatory mechanism was narrowed to the biosynthesis of unsaturated fatty acids signaling pathway, and it was predicted to be the most likely mechanism mediating SiNP-induced cytotoxicity. Overall, it is speculated that SiNP exposure alters the expression of stearoyl-CoA desaturase (SCD) and acyl-CoA thioesterase (ACOT), resulting in the over-synthesis of fatty acids and subsequently triggering apoptosis of trophoblast through the biosynthesis of unsaturated fatty acids signaling pathway.

### Molecular mechanism of SiNP-induced apoptosis of the HTR8 cells

**Fig. 4:**
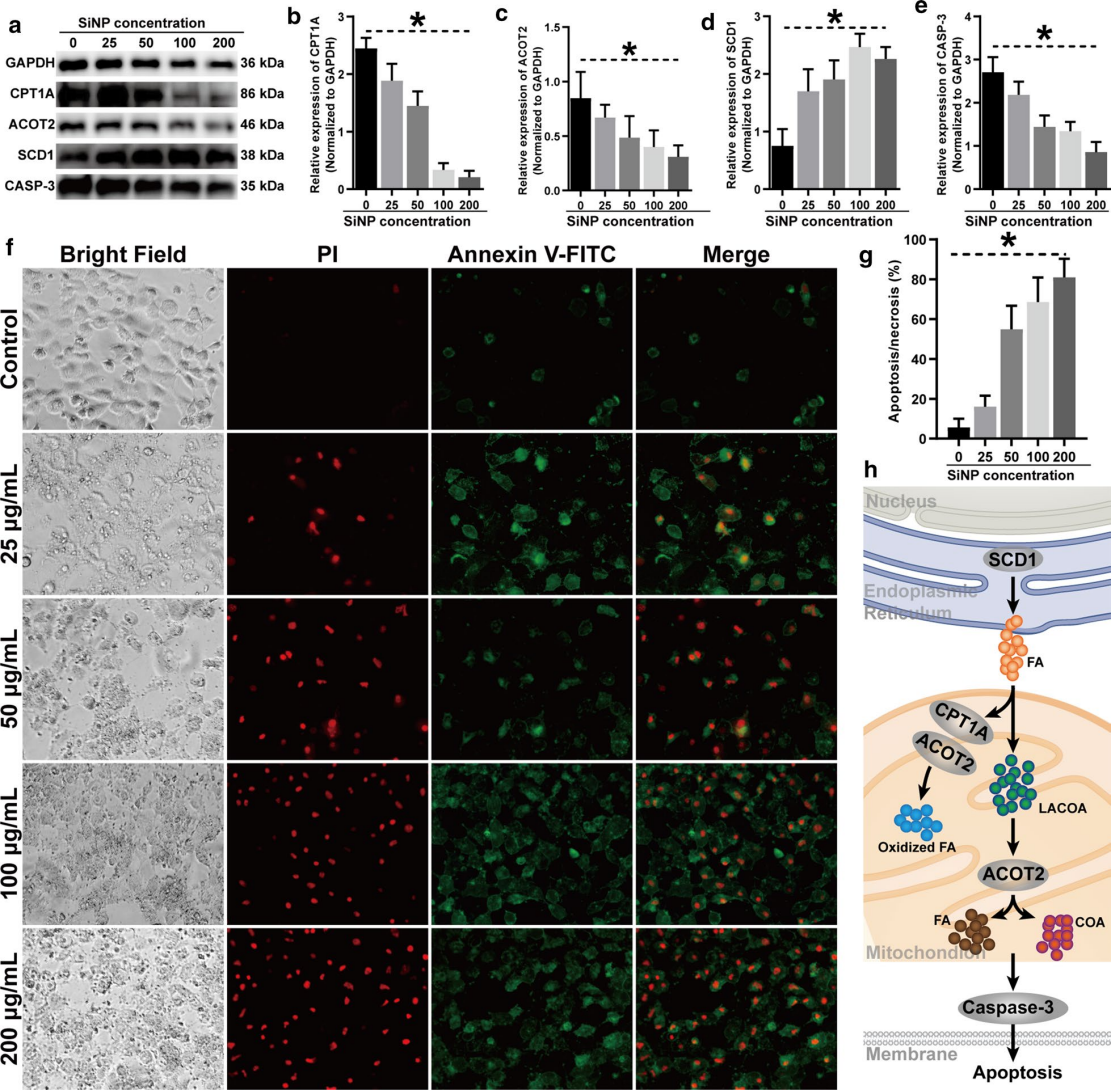
Pathogenesis of SiNP-induced HTR8 cell apoptosis. The HTR8 cells were exposed to SiNP at 25, 50, 100, 200 µg/mL for 24 hours, and the cells exposed to SiNP-free medium were used as controls. **a** Expression of Caspase-3, ACOT1, SCD1, and CPT1A detected by Western blot in the HTR8 cells. **b**–**e** Quantitative analysis of Caspase-3, ACOT1, SCD1, and CPT1A by normalizing to GAPDH, where the expression of Caspase-3, ACOT1 and CPT1A was negatively correlated with SiNP, but SCD1 showed positive correlationship. **f** Immunofluorescence images showing the apoptotic and necrotic HTR8 cells induced by SiNP exposure (×10 magnification). The apoptotic HTR8 cells were stained in green with Annexin V-FITC, and the necrotic HTR8 cells were stained in red with propidium iodide. **g** Quantitative analysis of the apoptotic and necrotic HTR8 cells in different groups. **h** Schematic illustration of the pathogenesis of SiNP-induced HTR8 cell apoptosis. The differences between different treatment groups were compared using one-way ANOVA followed by SNK post-hoc test. *P < 0.05

To investigate the association between the biosynthesis of unsaturated fatty acids and biological dysfunction of trophoblast, we detected the expression of acyl-coenzyme A thioesterase 2 (ACOT2), stearoyl coenzyme A desaturase 1 (SCD1), and carnitine palmitoyltransferase 1A (CPT1A) that were in charge of fatty acid biosynthesis. It was found that both ACOT2 and CPT1A were downregulated after SiNP exposure, whereas SCD1 was upregulated, and the alteration of these genes was consistent with the high throughput sequencing data (Fig. 4a–d). In addition, Caspase-3 was also detected as an indicator for mitochondria-dependent apoptosis, during which it could be divided into p17 and p19 subunits.[23] We found that the expression of Caspase-3 reduced in a dose-dependent manner (Fig. 4f), while p17 and p19 were upregulated (Additional file 1: Figure S2), suggesting the association between SiNP exposure and HTR8 cell apoptosis. Besides, the apoptotic and necrotic HTR8 cells were visualized by the dual fluorescence-staining method, and the number of both kinds of cells increased (Fig. 4f, g). Take the above findings into consideration, we depicted the pathogenesis of SiNP-induced trophoblast apoptosis in Fig. 4 h. In short, SiNP exposure alters the expression of SCD1, ACOT2, and CPT1A in the HTR8 cells, among which the upregulation of SCD1 induces over-synthesis of free fatty acids in the endoplasmic reticulum, while downregulation of ACOT2 and CPT1A suppresses fatty acids oxidization and long-chain acyl-coenzyme A dehydrogenase (LACoA) hydrolysis in mitochondria. The over-delivery of fatty acids from endoplasmic reticulum to mitochondria causes LACoA overload and subsequently leads to cell death through the biosynthesis of unsaturated fatty acids signaling pathway.

### Biodistribution and bioactivity of SiNP in vivo

**Fig. 5:**
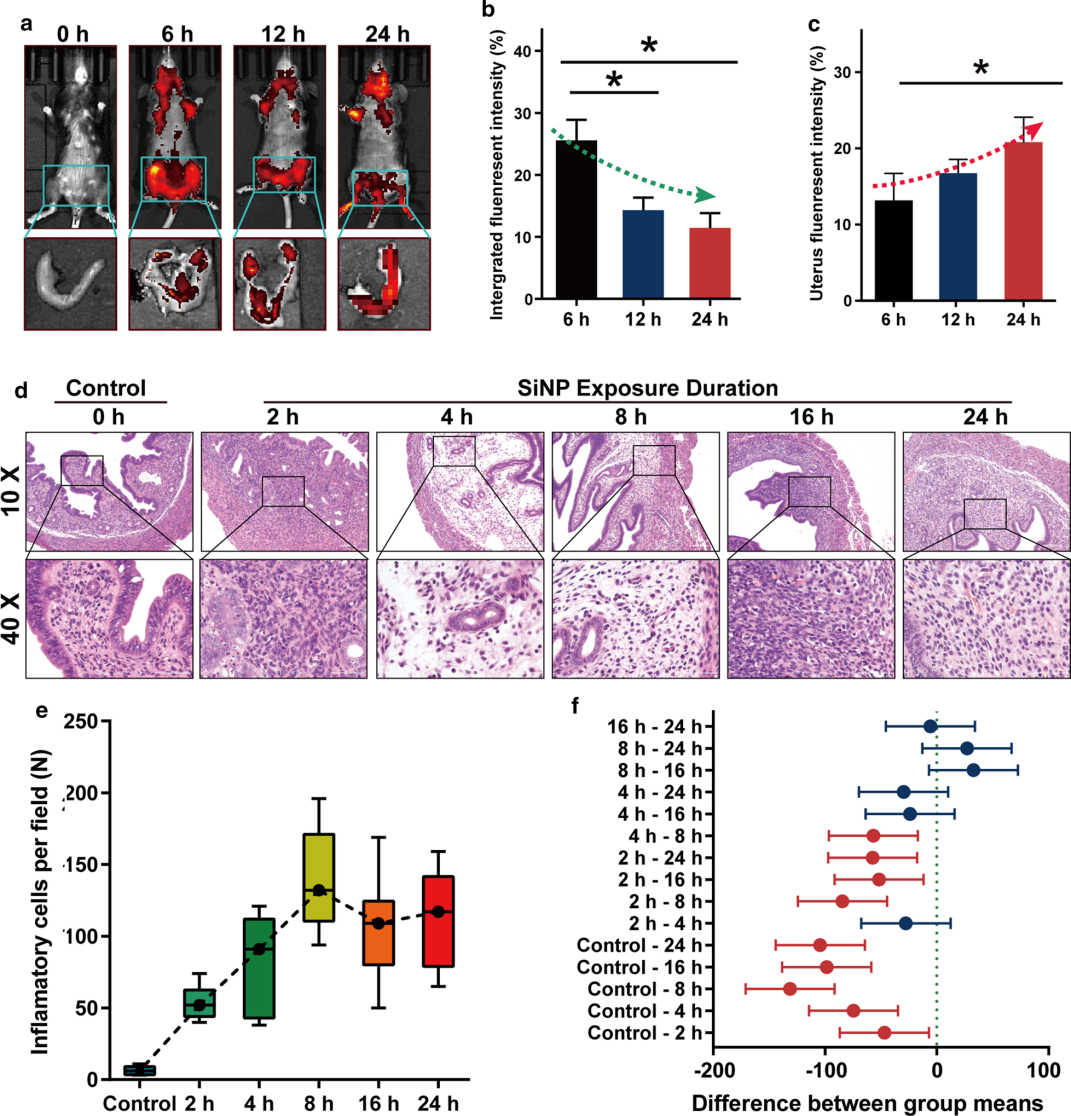
Biodistribution of SiNP in vivo and pathological changes of the uterus. The pregnant C57BL/6 mice were intratracheally instilled with 50 µL of FITC-SiNP at 5 mg/mL and observed using a live animal imaging system at 0, 4, 8, 16, 24 h. **a** Images showing the biodistribution and accumulation of FITC-SiNP in mice and their dissected uteri. **b**, **c** Quantitative analysis of the integrated fluorescence intensity at different observation time points, where it decreased as a whole in a time-depedent manner but increased in the uteri. **d** Images showing the pathological changes of the uteri. The infiltration of inflammatory cells exacerbated after SiNP exposure. **e** Boxplot showing the increased number of inflammatory cells in the uteri. **f** Forrest plot showing the comparison of inflammatory cells between different observation time points. The differences were compared using one-way ANOVA followed by SNK post-hoc test. *P < 0.05

**Fig. 6:**
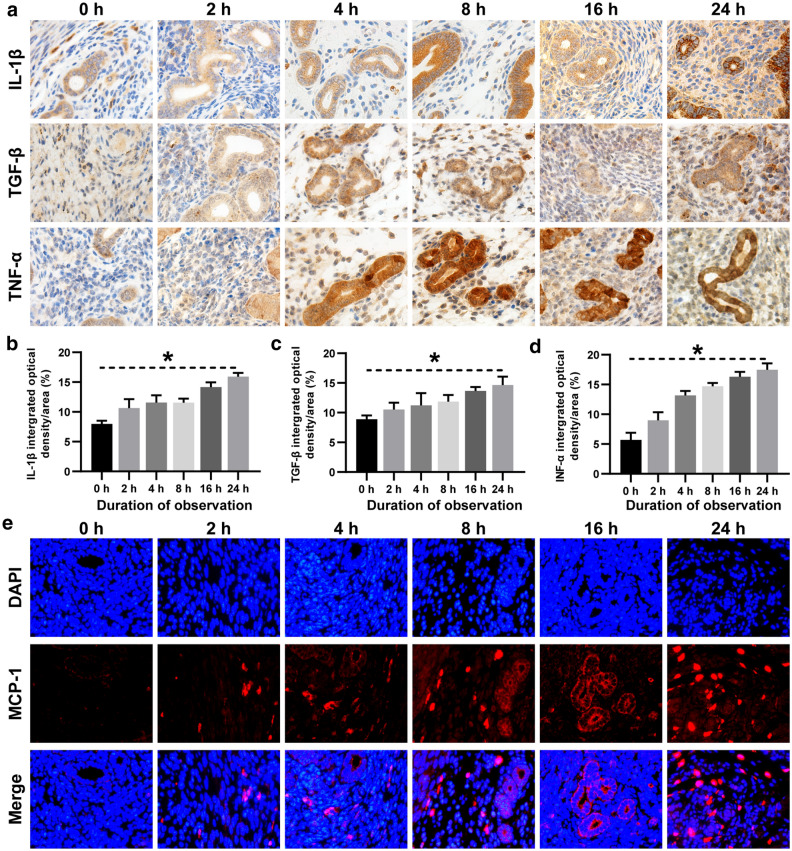
SiNP exposure elevated the expression of uterine cytokines in pregnant mice. **a** Immunohistochemical images showing the uterine expression of IL-1β, TGF-β, and TNF-α in mice administrated with 0.25 mg of SiNP (×20 magnification). The positive area of different cytokines was stained in brown. **b** Quantitative analysis showing the expression of IL-1β, TGF-β, and TNF-α at different observation time points. **c** Immunofluorescence images of uterine MCP-1 (×20 magnification). The nuclei were stained with DAPI in blue, and MCP-1 was stained with specific antibody in red. The differences were compared using one-way ANOVA followed by SNK post-hoc test. *P < 0.05

To track SiNP in vivo, we intratracheally instilled the FITC-SiNP into the C57BL/6 mice and observed them using the IVIS® Lumina III system. With the prolongation of observation duration, the chest fluorescence diminished gradually, but the uterine fluorescence strengthened (Fig. 5a–c). The uterine inflammatory cells were quanlified, and they immensely increased at the early stage of SiNP exposure (Fig. 5d), the hyperinflammatory status of the endometrium lasted to the end of this study (Fig. 5e–f). As shown in Fig. 6a–e, a time-dependent increase of interleukin-1α (IL-1α), transforming growth factor-β (TGF-β), tumor necrosis factor-α (TNF-α), and monocyte chemoattractant proteins-1 (MCP-1) was determined, and these cytokines were mainly secreted by the uterine macrophages (Additional file 1: Figure S3). Given the increased number of inflammatory cells and the elevated expression of inflammatory cytokines, we demonstrate that uterine accumulation of SiNP induces diffuse inflammatory infiltration of endometrium in the early stage of SiNP exposure.

### Pathogenesis of SiNP-induced uterine inflammation in vivo

**Fig. 7:**
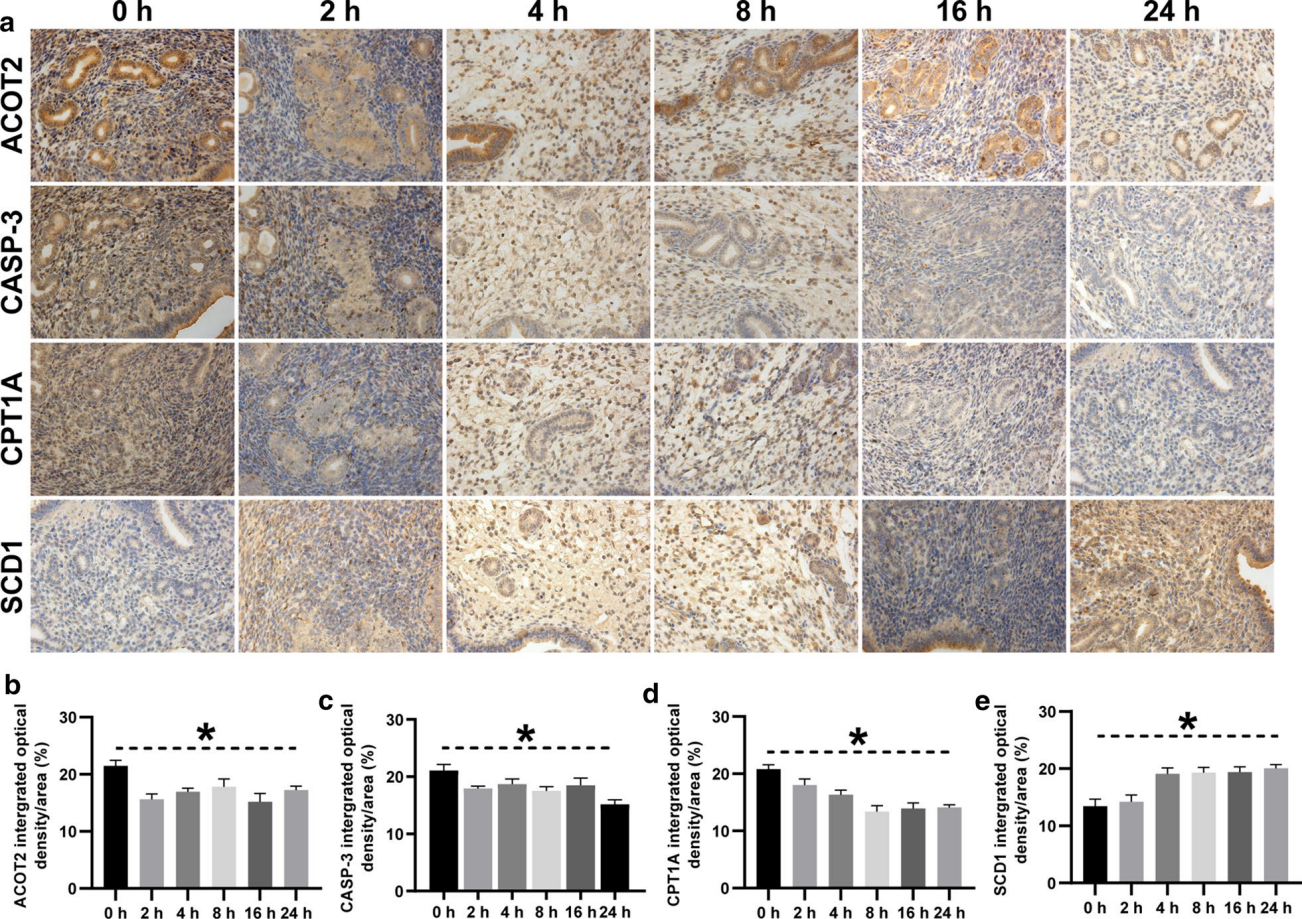
Uterine expression of genes associated with HTR8 cell apoptosis in vitro. **a** Immunohistochemical images showing the expression of SCD1, CPT1A, ACOT2, and Caspase-3 in vivo (×20 magnification). The positive area was stained in brown. **b**–**e** Quantitative analysis and comparison of ACOT2, Caspase-3, CPT1A, and SCD1 between different observation time points. Comparing with the uterine expression of specific genes at 0 h, the expression of CPT1A, ACOT2, and Caspase-3 increased at 2, 4, 8, 16, 24 h time points, whereas SCD1 decreased. The differences were compared using one-way ANOVA followed by SNK post-hoc test. *P < 0.05

Basing on the genome-wide alteration of gene expression, the molecular mechanism of SiNP-induced uterine inflammation in vitro was previously investigated using the bioinformatics analysis, where we found the dysregulation of SCD1, CPT1A, ACOT2, and Caspase-3 significantly changed the content of fatty acid, and therefore we detected them in mice exposed to SiNP in vivo. As shown in Fig. 7a–e, the expression of CPT1A, ACOT2, and Caspase-3 decreased as compared to the control animals, whereas SCD1 increased. Noteworthily, the alteration of these genes between 2 and 24 h was of no statistical significance, suggesting that they were sensitive indicators for the early-stage exposure of SiNP. Given the uterine inflammatory infiltration and dysregulation of SCD1, CPT1A, and ACOT2, these results confirmed the association between SiNP exposure and uterine inflammation.

## Discussion

By coupling with FITC, the FITC-SiNP was chemically synthesized and applied to track SiNP in vitro and in vivo. First, the chemical-physical characteristics of FITC-SiNP were studied, and they were highly identical with nanoparticles obtained in previous studies [24]. Besides, the FITC-SiNP was highly consistent with SiNP in monodispersity and stability, which provided an efficient approach for localizing SiNP in future studies.

To evaluate the biological effect of SiNP on reproduction in vitro, we treated the HTR8 cells with FITC-SiNP and confirmed the internalization of these fluorescent nanoparticles. Previously, it was reported that the intravenously administrated SiNP could translocate to placenta [7], and the orally administered SiNP was detected to be higher in liver and ovary than that in brain, kidney, and spleen [25]. Notably, the detrimental effects of SiNP internalization were reported on a variety of cells, such as sperms, Leydig cells, and human umbilical vein endothelial cells [26]. However, seldom studies, if any, have investigated the effects of SiNP on trophoblast. It is common sense that trophoblast plays an indispensable role in placenta formation, embryo implantation, and fetal development, and we find adverse effects of SiNP exposure on the related biological processes, including cell migration, invasion, and tube formation. Consistently, SiNP was demonstrated to block the angiogenesis of bone marrow-derived rat mesenchymal stem cells and suppress tissue repair [27]. However, the inflammatory factors secreted by the SiNP-exposed dendritic cells were found to promote angiogenesis [28]. For nanoparticles derived from carbon-based nanomaterials, they were also found to inhibit vasculogenesis and angiogenesis in zebrafish and chicken embryos [29]. In particular, exposure to carbon nanoparticles was potentially associated with rat infertility [30]. Given the detrimental effects of SiNP on HTR8 cells, we showed the preliminary evidence for the increased infertility and abortion in areas with severe air pollution.

The high throughput sequencing and bioinformatics analysis was adopted to predict the underlying mechanism of SiNP-induced trophoblast dysfunction, from which a total of six signaling pathways were archived. In view of data published in the literature, exposure to TiO_2_ or Ag nanoparticles could activate the pentose phosphate in plants [31, 32], but no available evidence was determined in animals or human beings. Similarly, Ag nanoparticle exposure was found to dysregulate cysteine and methionine metabolism and led to liver damage [33]. Importantly, the AMP-activated protein kinase (AMPK) signaling pathway could promote cell toxicity in SiNP-exposed spermatocytes [34]. However, the role of peroxisome proliferator activated receptor (PPAR) and unsaturated fatty acids signaling pathways have not been revealed in SiNP-exposed trophoblast.

Furthermore, a series of experiments were adopted to reveal the regulatory pattern of the biosynthesis of unsaturated fatty acids signaling pathway in response to SiNP exposure. The transcriptional alteration of the dysregulated genes such as ACOT2, CPT1A, and SCD1 was in line with that at the protein level. Functionally, ACOT2 was responsible for hydrolyzing LACoA into free fatty acids and CoA and cooperatively oxidizing fatty acids with CPT1A (Fig. 4 h) [35, 36]. In contrast, SCD1 was essential for the biosynthesis of fatty acids [37]. Therefore, the inhibition of ACOT2 and CPT1A promoted LACoA accumulation, while upregulation of SCD1 led to the over-synthesis of fatty acid. However, excessive fatty acid exacerbated LACoA accumulation, thereby resulting in mitochondrial overload of metabolic wastes and even trophoblast apoptosis. Consistently, it had been reported that SiNP exposure triggered apoptosis of granulosa cells and induced follicular atresia in BABL/c mice [38]. In particular, the 20 nm-sized SiNP had been proven to cause oxidative stress and apoptosis of cells in the uterine connective tissues, which could damage the contractile activity [6]. For in vivo studies, SiNP was found to translocate to the placenta of mice and deposite in the fetuses during pregnancy, and the major impact of SiNP exposure was DNA damage in offspring [7, 17]. The orally administrated Ag nanoparticles were also detected in the uterus of experimental animals and induced reproductive and developmental toxicity [39].

For the the association between FA dysregulation, cell death, and inflammation, it had been preliminarily revealed by previous studies. For example, adiponectin, a fat-derived hormone, was demonstrated to stimulate AMPK and cyclooxygenase-2 activities, both of which could antagonize apoptosis and TNFα production, thereby suppressing tissue inflammation in heart diseases [40]. Lipid accumulation was reported to generate toxic effects, leading to hepatocellular damage and inflammation through triggering cell apoptosis via endoplasmic stress [41]. In particular, Ag nanoparticle was confirmed to cause Aβ deposition in neuronal cells, and subsequently enhance the secretion of MCP-1 and IL-6 and induce neuronal cell apoptosis, and these biological alterations finally promoted the pathogenesis of Alzheimer’s disease [42]. In this study, we conducted high through put sequencing analysis in trophoblast exposed to SiNP and found that the apoptosis of trophoblast induced uterine inflammation. However, a certain number of the dysregulated genes were also overlapped with genes in the classical inflammatory pathways. As shown in Additional file 1: Figure S4, three intersections exhibited the number of dysregulated genes in different inflammatory signaling pathways. In combination with data obtained in this study, we demonstrated the association between SiNP-induced FA dysregulation, cell death, and inflammation.

## Conclusions

By coupling with FITC, SiNPs are labeled with fluorescence and are tracked in vitro and in vivo. The FITC-SiNP is visualized in the HTR8 cells, and the internalization of SiNP causes trophoblast apoptosis and biological dysfunction, which are associated with uterine inflammation in vivo. Mechanistically, exposure to SiNP dysregulates 75 genes in trophoblast, among which the dysregulation of ACOT2, SCD1, and CPT1A is demonstrated to induce LACoA overload and cell apoptosis. Overall, the chemical conjugation of FITC onto SiNP provides a practical approach for tracking SiNP in vitro and in vivo, and exposure to SiNP induces uterine inflammation through triggering trophoblast apoptosis, which is mediated by the ACOT/CPT1A/SCD1 axis act through the biosynthesis of unsaturated fatty acids signaling pathway. Findings obtained in this study would contribute to a comprehensive understanding of the biological effects of SiNP as well as the formulation of environmental measures.

## Materials and methods

### Materials

SiNPs (spherical, porous, 5–20 nm particle size under the transmission electron microscope (TEM)) were obtained from Sigma-Aldrich (MO, USA). FITC was bought from Solarbio (Beijing, China). Tubulin Tracker™ Deep Red was obtained from Thermo Fisher (MA, USA). Cell count kit-8 (CCK8), Annexin V-FITC cellular apoptosis detection kit, Hoechst staining kit, DAPI, and BeyoClick™ Edu-488 kit were purchased from Beyotime (Beijing, China). Antibodies, including Caspase-3, ACOT2, SCD1, CPT1A, GAPDH, and goat anti-rabbit IgG, were obtained from Abcam (Cambridge, UK). Fetal bovine serum (FBS) and Dulbecco’s modified Eagle medium (DMEM) were provided by Hyclone (IL, USA). The human trophoblast cell line HTR8 was purchased from the American Type Culture Collection (ATCC) (VA, USA), and the cells were derived by transfecting the cells that grew out of chorionic villi explants of human first-trimester placenta.

### Chemical synthesis and characterization of FITC-SiNP

FITC-SiNP was chemically synthesized based on protocols previously published [24, 43]. In brief, we coupled FITC onto SiNP in two steps. The first step was to modify NH_2_ onto the surface of SiNP, where a mixture of 2 mL of 3-aminopropyltriethoxysilane (APTS) and 100 mL of anhydrous dry toluene was prepared in advance, and 1.0 g of SiNP was refluxed for 24 h. Subsequently, the mixture was filtered to obtain the raw nanoparticles and then purified by rinsing with distilled water and methanol and desiccated at 80 °C under the high vacuum for 12 h. The purified SiNP was ammonized to form the APTES-modified SiNPs (NH_2_-SiNPs). Secondly, the NH_2_-SiNPs were labeled with fluorescein. A mixture of 2.5 mg of FITC, 100 µL of APTES, and 3 mL of ethanol was prepared and vibrated in the dark overnight. Then, 200 mL of N-cetyltrimethylammonium bromide (CTAB) solution at 225 mg/mL was prepared and heated up to 80°C, and a moderate amount of sodium hydroxide was added by the concentration of 2 M. After the mixture being stirred thoroughly, 3 mL tetraethylorthosilicate (TEOS) was added to the heated mixture drop by drop, and 250 µL of NH_2_-SiNP solution was added in turn. Finally, the newly generated mixture was stirred for 2 h to obtain the precipitates which were subjected to the acidified methanol solution to harvest the FITC-SiNPs.

The chemical-physical characteristics of SiNP and FITC-SiNP were examined according to previous studies [24, 44, 45]. The morphological characteristics of SiNP and FITC-SiNP were studied using SEM (Carl Zeiss, Oberkochen, Germany), and the fluorescence of FITC-SiNP was observed using fluorescence microscopy (OLYMPUS, Tokyo, Japan). The characteristics of SiNP and FITC-SiNP in pure water and DMEM were studied using ZetaView (Particle Metrix, Inning am Ammersee, Germany), and the particle FTIR spectra (4000–500 cm^− 1^) in KBr were measured using a spectrometer (Thermo Fisher, MA, USA). The structural property of SiNP was analyzed using XRD (XRD, Shimadzu LabX XRD-6000).

### Cell culture and intervention

The human trophoblast cell line HTR8 cells were seeded at 1.5 × 10^4^/well and maintained in a standard carbon dioxide incubator with a humidified atmosphere at 37°C and 5 % CO_2_. The complete culture medium was prepared by adding 10 % FBS to DMEM, and the HTR8 cells were incubated at 80 % confluency 24 h later, then they were exposed to SiNP at 25, 50, 100, 200 µg/mL. Those HTR8 cells maintained in SiNP-free medium were used as controls.

### Cell viability assay

A total of 1 × 10^4^ HTR8 cells/well were seeded in a 96-well plate and exposed to SiNP at 0, 25, 50, 100, and 200 µg/mL. After incubation for 24 h, the culture medium was refreshed with SiNP-free medium, followed by incubation for 24 h, and stained with 20 µL of CCK8 solution/well. Two hours later, the absorbance of each well was recorded at 460 nm. Ten samples were repeated in parallel for each group.

### SiNP uptake assay in vitro

According to the detection result of CCK8 assay, the median lethal dose (LD_50_) of SiNP on trophoblast was determined as 100 µg/mL and was used to treat the HTR8 cells for 2 h. After rinsing the cells with PBS, the cytoskeleton was stained with Docetaxel for 30 min, and the nuclei were stained with Hoechst solution for 30 min. Then, the internalization of FITC- SiNP by the HTR8 cells was observed using ImageXpress Micro Confocal system (Molecular Devices, California, USA), and three absorbance spectra of 364 nm, 490 nm, and 652 nm were applied to visualize the nuclei, SiNPs, and cell skeleton.

### Cell proliferation assay

A total of 1.2 × 10^6^ HTR8 cells/well were seeded in a 6-well plate and exposed to SiNP at 0, 25, 50, 100, and 200 µg/mL. After incubation for 24 h, the culture medium was replaced with fresh DMEM mixed with EdU solution. Two hours later, the medium was discarded, and the cells were fixed with 4 % paraformaldehyde for 15 min at room temperature and penetrated using Triton X-100 for 10 min. Then, 100 µL of 4′,6-diamidino-2-phenylindole (DAPI) solution was added to stain the nuclei for 20 min. Finally, the cells were subjected to the ImageXpress Micro Confocal system (Molecular Devices, California, USA), and two absorbance spectra of 364 nm and 595 nm were applied to visualize the nuclei and proliferative cells.

### Cell apoptosis assay

In line with protocols described above, 1.5 × 10^4^ HTR8 cells/well were exposed to SiNP at 0, 25, 50, 100, and 200 µg/mL for 24 h. Then, the apoptotic cells were detected using the Hoechst staining kit and Annexin V-FITC apoptosis detection kit. First, the HTR8 cells were stained with 0.5 mL of Hoechst 33,324 for 20 min and observed using the ImageXpress Micro Confocal system at 350 nm wavelength. Alternatively, 5 µL of Annexin V-FITC and 10 µL of propidium iodide (PI) were added to the HTR8 cell in turn, and the cells were incubated in the dark at room temperature for 10 min. The apoptotic and necrotic cells were detected using the ImageXpress Micro Confocal system at absorbance spectra of 488 nm and 535 nm.

### Wound healing assay

The protocol for wound healing assay was previously described [46]. In brief, a total of 1.2 × 10^6^ HTR8 cells/well were seeded onto a 6-well plate and exposed to SiNP at 0, 25, 50, 100, and 200 µg/mL for 24 h. A wound was scraped into the middle region of the cell layer with a plastic tip of 100 µL, and then the plate was washed with PBS 3 times to remove the unattached cells. After incubation for 24 h, the wounds were observed with an optical microscopy (Olympus, Tokyo, Japan), and the area of the scratches was measured. The wound healing ability of the HTR8 cells was assessed by the reduced area of the wound.

### Tube formation assay

An equal volume of 100 mL of chilled liquid Matrigel (Corning, Michigan, USA) was added to each well of a 96-well plate and kept at 37 °C for 30 min. Then, 1 × 10^4^ HTR8 cells were suspended in 100 µL of DMEM and seeded onto a 96-well plate. After incubation for 6 h, the HTR8 cells were observed using an optical microscope, and the total length of tubes was measured using the Angiogenesis Analyzer package embedded in ImageJ version 1.52a for Windows (NIH, USA). The tube formation ability was evaluated by measuring the total length of the tubes. Each treatment group was repeated with 5 samples in parallel.

### High throughput sequencing and bioinformatics analysis

A total of 1.2 × 10^6^ HTR8 cells/well were seeded in a 6-well plate and exposed to SiNP-free DMEM or SiNP at 100 µg/mL. After incubation for 24 h, the total mRNA of the HTR8 cells was extracted and profiled using Illumina HiSeq 2500 (California, USA). Limma analysis was conducted to identify the aberrantly expressed genes according to the preset conditions: adjusted *P*-value < 0.05 and the absolute value of log_2_(Fold Change) ≥ 1. The clusterProfiler version 3.14.3, an R package, was applied for the bioinformatics analysis, and it was adopted from two aspects of Gene Ontology (GO) analysis and Kyoto Encyclopedia of Genes and Genomes (KEGG) analysis. A pipeline illustrating the strategy for bioinformatics analysis was shown in Additional file 1: Figure S5.

### Western blot assay

A total of 1.2 × 10^6^ HTR8 cells/well were seeded in a 6-well plate and exposed to SiNP at 0, 25, 50, 100, and 200 µg/mL, and the cells were harvested after incubation for 24 h. The total protein was extracted by lysing the cells on ice for 30 min and then quantified using the BCA kit, among which 20 µg of protein were separated and transferred onto the PVDF membranes (Millipore, Billerica, MA) via the sodium dodecyl sulfate-polyacrylamide gel electrophoresis (SDS-PAGE). The PVDF membranes were blocked in 5 % nonfat milk at 4°C for 2 h, and then incubated with the rabbit anti-human primary antibodies of Caspase-3, ACOT2, SCD1, CPT1A, and GAPDH overnight. After rinsing with TBST 3 times, the membranes were incubated with the goat anti-rabbit IgG for 2 h. The primary antibodies were diluted at 1:1000, and the secondary antibody was 1:3000. Finally, all membranes were subjected to the Fluor Chem HD2Gel imaging system (Proteinsimple, CA, USA), and the relative expression of the target protein was quantified by normalizing to GAPDH.

### Biodistribution of SiNP in vivo

The review board of the Maternal and Child Health Care Hospital of Shandong Province Affiliated to Shandong University approved this study (SDSFY-ETH-001). A total of 36 C57BL/6 female mice were obtained from Laboratory Animal Resources, Chinese Academy of Sciences (Beijing, China), and kept in a pathogen-free animal facility with standard maintenance conditions at Zhengzhou University. Based on previous studies conducted to simulate occupational silica dust exposure and develop animal silicosis model [47], 30 mice were intratracheally instilled with 50 µL of FITC-SiNPs at 5 mg/mL when they grew up to 6–8 weeks of age, weighing approximately 25–30 g. In parallel, 6 mice in the control group were instilled with the same volume of normal saline. The translocation of FITC-SiNP in vivo was tracked using the IVIS® Lumina III system (PerkinElmer, Massachusetts, USA), and the biodistribution of FITC-SiNP was captured at 0, 6, 12, and 24 h. Meanwhile, the mouse uteri were separated, on which the fluorescence was examined.

### Histopathology examination

After instilling 50 µL of SiNP, the mice were sacrificed at 0 h, 2, 4, 8, 16, and 24 h, 6 mice per time point. The mice uteri were separated and fixed in 4 % paraformaldehyde and stained with hematoxylin and eosin (HE). The histopathological changes were examined using an optical microscope, and the inflammatory cells were quantified and compared between different observation durations.

### Immunohistochemistry and immunofluorescence

The mice were instilled with 50 µL of SiNP and sacrificed at 0 h, 2, 4, 8, 16, and 24 h, from which the uteri were isolated, fixed, and embedded. For immunohistochemistry, the uterine sections were incubated with specific primary antibodies, including TNF-α, TGF-β, IL-1β, SCD1, CPT1A, ACOT1, and Caspase-3. Alternatively, the uterine sections were incubated with MCP-1 by immunofluorescence. The protein expression level was quantified by Image-Pro Plus version 6.0 (Media Cybernetics, Maryland, USA).

### Statistics

All data were analyzed and visualized using SAS version 9.4 for Windows (SAS Institute Inc., Cary, NC, USA). The differences between different treatment groups were compared using the one-way analysis of variance (ANOVA) followed by SNK post-hoc test. The differentially expressed genes were identified using Limma test. Principal component analysis (PCA) and Pearson correlation analysis were adopted to investigate the homogeneity of samples in different groups. Clustering analysis was applied to visualize samples and dysregulated genes. A *P*-value of less than 0.05 was considered statistically significant unless otherwise indicated.

## Supplementary Information

**Additional file 1.** Figure S1. XRD pattern of silica nanoparticles. XRD, X-ray diffraction. Figure S2. Expression (A) and comparison of p17 and p19 (B & C) between trophoblasts with different interventions. Figure S3. Dual staining of uterine macrophage and cytokines. The macrophage was stained with F4/80 antibody in red, the cytokines were labeled with TNF-α, TGF-β, and IL-1β antibodies in green, and the nuclei were stained with DAPI in blue. The relative distribution of macrophage and cytokines were visualized in the merged and enlarged panels. Figure S4. Venn diagram exhibiting the dysregulated genes associated with inflammation. DEGs, differentially expressed genes. MAPK, Mitogen-activated protein kinase 1. JAK, Janus kinase. NF-κB, Nuclear factor-κB. Figure S5. Pipeline of the high throughput sequencing and bioinformatics analysis. Table S1. Hydrodynamic size, zeta potential, and polydispersity index of SiNP and FITC-SiNP measured by DLS in ultrapure water and DMEM with 10%FBS. Table S2. The Differentially Expressed Genes in Trophoblast Exposed to Silica Nanoparticles. Table S3. Signaling Pathway Enrichment Analysis for Dysregulated Genes in Trophoblast Exposed to Silica Nanoparticles.

## Data Availability

All data analyzed during the current study are available from the corresponding author upon request.
