# Supplementary material for: Uterine metabolic disorder induced by silica nanoparticles: biodistribution and bioactivity revealed by labeling with FITC
Source: J Nanobiotechnology. 2021 Feb 28;19:62. doi: 10.1186/s12951-021-00810-x (PMC7916316; doi:10.1186/s12951-021-00810-x)
Supplement: Supplementary file 1 — Additional file 1. Figure S1. XRD pattern of silica nanoparticles. XRD, X-ray diffraction. Figure S2. Expression (A) and comparison of p17 and p19 (B & C) between trophoblasts with different interventions. Figure S3. Dual staining of uterine macrophage and cytokines. The macrophage was stained with F4/80 antibody in red, the cytokines were labeled with TNF-α, TGF-β, and IL-1β antibodies in green, and the nuclei were stained with DAPI in blue. The relative distribution of macrophage and cytokines were visualized in the merged and enlarged panels. Figure S4. Venn diagram exhibiting the dysregulated genes associated with inflammation. DEGs, differentially expressed genes. MAPK, Mitogen-activated protein kinase 1. JAK, Janus kinase. NF-κB, Nuclear factor-κB. Figure S5. Pipeline of the high throughput sequencing and bioinformatics analysis. Table S1. Hydrodynamic size, zeta potential, and polydispersity index of SiNP and FITC-SiNP measured by DLS in ultrapure water and DMEM with 10%FBS. Table S2. The Differentially Expressed Genes in Trophoblast Exposed to Silica Nanoparticles. Table S3. Signaling Pathway Enrichment Analysis for Dysregulated Genes in Trophoblast Exposed to Silica Nanoparticles. [file 12951_2021_810_MOESM1_ESM.docx]

**Uterine metabolic disorders induced by silica nanoparticles: biodistribution and bioactivity revealed by labeling with FITC**

Shuyin Duan^a, b^ Meihua Zhang^a^, Junxia Li^c^, Jiaqi Tian^c^, Haoyu Yin^c^, Xietong Wang^a, d^, Lin Zhang^*a^

^a^ Key Laboratory of Birth Regulation and Control Technology of National Health Commission of China, Maternal and Child Health Care Hospital of Shandong, Shandong University, Jinan 250001, China.

^b^ School of Public Health, Zhengzhou University, Zhengzhou 450001, China.

^c^ School of Public Health, Weifang Medical University, Weifang 261053, China.

^d^ Department of Obstetrics and Gynecology, Shandong Provincial Hospital, Jinan 250001, China.

**^*^Correspondence to:** Lin Zhang, Ph.D., M.D.

Key Laboratory of Birth Regulation and Control Technology of National Health Commission of China, Maternal and Child Health Care Hospital of Shandong, Shandong University, Jinan 250001, China. Tel: +86-18668959905, E-mail: zhanglin8901@163.com

**Additional Figures**

**Figure S1.**


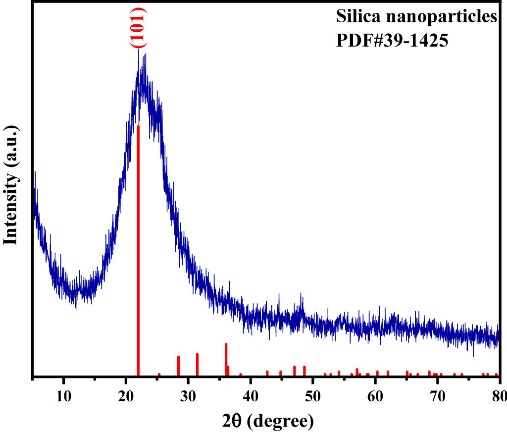


**Figure S1.** XRD pattern of silica nanoparticles. XRD, X-ray diffraction.

**Figure S2.**


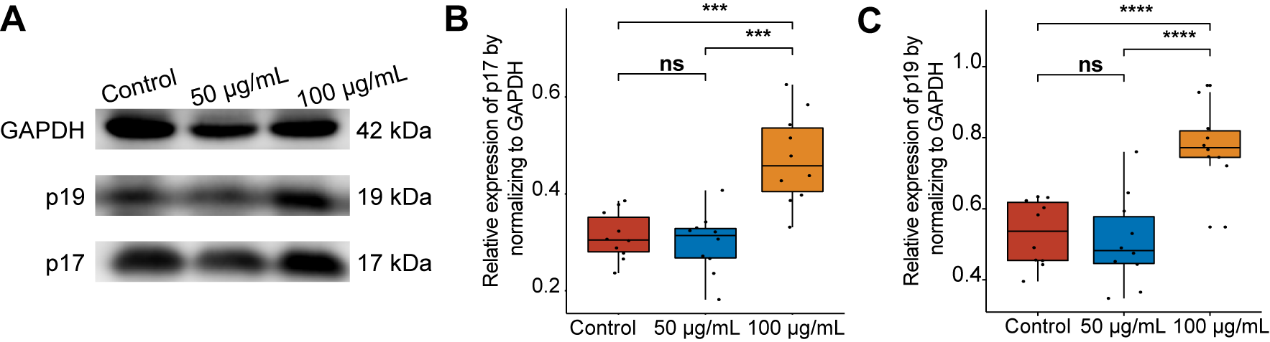


**Figure S2.** Expression (A) and comparison of p17 and p19 (B & C) between trophoblasts with different interventions.

**Figure S3.**

**
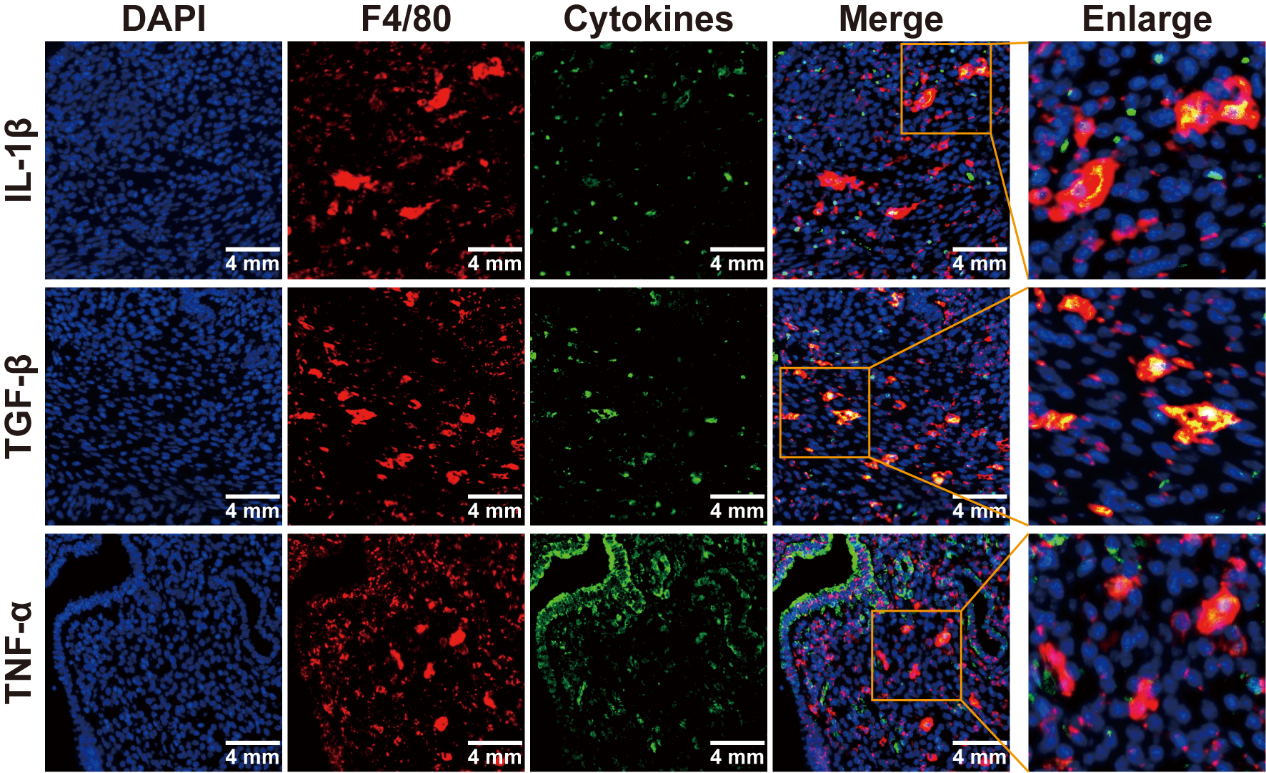
**

**Figure S3.** Dual staining of uterine macrophage and cytokines. The macrophage was stained with F4/80 antibody in red, the cytokines were labeled with TNF-α, TGF-β, and IL-1β antibodies in green, and the nuclei were stained with DAPI in blue. The relative distribution of macrophage and cytokines were visualized in the merged and enlarged panels.

**Figure S4.**


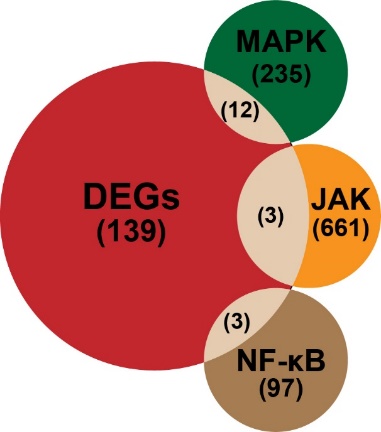


**Figure S4.** Venn diagram exhibiting the dysregulated genes associated with inflammation. DEGs, differentially expressed genes. MAPK, Mitogen-activated protein kinase 1. JAK, Janus kinase. NF-κB, Nuclear factor-κB.

**Figure S5.**

**

**

**Figure S5.** Pipeline of the high throughput sequencing and bioinformatics analysis

**Additional Tables**

**Table S1.** Hydrodynamic size, zeta potential, and polydispersity index of SiNP and FITC-SiNP measured by DLS in ultrapure water and DMEM with 10%FBS

| **Samples** | **Size (nm)** | | **ζ-Potential (mV)** | | **Polydispersity Index (PDI)** | |
| --- | --- | --- | --- | --- | --- | --- |
|  | Ultrapure Water | DMEM-10%FBS | Ultrapure Water | DMEM-10%FBS | Ultrapure Water | DMEM-10%FBS |
| **SiNP** | 35.7 ± 4.8 | 58.1 ± 10.3 | -31.2 ± 0.3 | -11.4 ± 0.6 | 0.13 | 0.19 |
| **FITC-SiNP** | 50.2 ± 5.6 | 126.7 ± 18.2 | -17.7 ± 0.2 | -9.1 ± 0.3 | 0.11 | 0.21 |

**Table S2.** The Differentially Expressed Genes in Trophoblast Exposed to Silica Nanoparticles

| ID | Symbols | log_2_(Fold Chane) | t Value | B Value | P Value | Adjusted P Value | Regulation |
| --- | --- | --- | --- | --- | --- | --- | --- |
| 1 | Gm10471 | 2.95 | 15.92 | 6.52 | 2.55E-07 | 0.001 | Up |
| 2 | Fam107a | 2.79 | 6.22 | 0.97 | 2.59E-04 | 0.048 | Up |
| 3 | Pou2af1 | 2.73 | 9.06 | 3.39 | 1.81E-05 | 0.017 | Up |
| 4 | Rln1 | 2.55 | 6.49 | 1.24 | 1.94E-04 | 0.044 | Up |
| 5 | Cyp2f2 | 2.18 | 8.68 | 3.11 | 2.49E-05 | 0.019 | Up |
| 6 | Tfap2c | 2.15 | 5.96 | 0.70 | 3.44E-04 | 0.048 | Up |
| 7 | Fbxo15 | 2.12 | 6.16 | 0.91 | 2.77E-04 | 0.048 | Up |
| 8 | Scgn | 2.06 | 6.79 | 1.53 | 1.43E-04 | 0.041 | Up |
| 9 | Gipr | 2.01 | 6.18 | 0.93 | 2.70E-04 | 0.048 | Up |
| 10 | Bmp8a | 1.79 | 6.69 | 1.44 | 1.57E-04 | 0.043 | Up |
| 11 | Cbs | 1.76 | 7.62 | 2.28 | 6.36E-05 | 0.027 | Up |
| 12 | Pnma2 | 1.66 | 6.11 | 0.86 | 2.90E-04 | 0.048 | Up |
| 13 | 4930583H14Rik | 1.58 | 6.91 | 1.65 | 1.26E-04 | 0.038 | Up |
| 14 | Cpa1 | 1.56 | 7.31 | 2.01 | 8.53E-05 | 0.031 | Up |
| 15 | Il1r2 | 1.46 | 6.25 | 1.00 | 2.50E-04 | 0.048 | Up |
| 16 | Bcat1 | 1.40 | 6.55 | 1.30 | 1.82E-04 | 0.044 | Up |
| 17 | 9230102K24Rik | 1.39 | 6.45 | 1.20 | 2.03E-04 | 0.045 | Up |
| 18 | Hsd11b1 | 1.29 | 6.70 | 1.45 | 1.56E-04 | 0.043 | Up |
| 19 | E2f1 | 1.22 | 7.86 | 2.48 | 5.08E-05 | 0.025 | Up |
| 20 | Dlk1 | 1.17 | 7.21 | 1.92 | 9.40E-05 | 0.032 | Up |
| 21 | Gm14327 | 1.16 | 5.96 | 0.69 | 3.46E-04 | 0.048 | Up |
| 22 | Rbp1 | 1.12 | 6.51 | 1.26 | 1.90E-04 | 0.044 | Up |
| 23 | Bnip3 | 1.11 | 7.70 | 2.35 | 5.88E-05 | 0.027 | Up |
| 24 | Tmem151b | 1.11 | 6.04 | 0.79 | 3.14E-04 | 0.048 | Up |
| 25 | 2810417H13Rik | 1.10 | 6.27 | 1.02 | 2.45E-04 | 0.047 | Up |
| 26 | Etv4 | 1.10 | 6.01 | 0.75 | 3.27E-04 | 0.048 | Up |
| 27 | Gm14207 | 1.09 | 5.95 | 0.68 | 3.49E-04 | 0.048 | Up |
| 28 | Pla1a | 1.07 | 6.77 | 1.51 | 1.45E-04 | 0.041 | Up |
| 29 | Uhrf1 | 1.07 | 6.41 | 1.16 | 2.11E-04 | 0.045 | Up |
| 30 | Glipr2 | 1.06 | 6.12 | 0.86 | 2.89E-04 | 0.048 | Up |
| 31 | Lig1 | 1.04 | 6.21 | 0.96 | 2.62E-04 | 0.048 | Up |
| 32 | Stk32c | 1.03 | 6.31 | 1.06 | 2.34E-04 | 0.046 | Up |
| 33 | Adck2 | -1.00 | -6.42 | 1.17 | 2.09E-04 | 0.045 | Down |
| 34 | Slc27a1 | -1.01 | -6.63 | 1.38 | 1.68E-04 | 0.044 | Down |
| 35 | Acacb | -1.03 | -7.59 | 2.25 | 6.55E-05 | 0.027 | Down |
| 36 | 1300010F03Rik | -1.08 | -9.25 | 3.52 | 1.56E-05 | 0.017 | Down |
| 37 | Lipe | -1.08 | -8.52 | 3.00 | 2.84E-05 | 0.020 | Down |
| 38 | Slc25a20 | -1.11 | -9.06 | 3.38 | 1.83E-05 | 0.017 | Down |
| 39 | Retsat | -1.14 | -9.73 | 3.83 | 1.08E-05 | 0.015 | Down |
| 40 | Gm17251 | -1.17 | -8.01 | 2.60 | 4.43E-05 | 0.024 | Down |
| 41 | Nkain1 | -1.17 | -7.67 | 2.32 | 6.06E-05 | 0.027 | Down |
| 42 | Adra1b | -1.18 | -9.86 | 3.91 | 9.78E-06 | 0.015 | Down |
| 43 | Paqr9 | -1.20 | -7.40 | 2.09 | 7.79E-05 | 0.028 | Down |
| 44 | Mlycd | -1.23 | -9.47 | 3.66 | 1.31E-05 | 0.017 | Down |
| 45 | Hsdl2 | -1.36 | -12.06 | 5.10 | 2.14E-06 | 0.005 | Down |
| 46 | Plbd1 | -1.37 | -8.39 | 2.90 | 3.18E-05 | 0.020 | Down |
| 47 | Ucp2 | -1.37 | -7.43 | 2.12 | 7.58E-05 | 0.028 | Down |
| 48 | Mtl5 | -1.38 | -7.01 | 1.75 | 1.14E-04 | 0.035 | Down |
| 49 | Nwd1 | -1.43 | -8.45 | 2.94 | 3.02E-05 | 0.020 | Down |
| 50 | Cpt1a | -1.49 | -10.59 | 4.34 | 5.74E-06 | 0.010 | Down |
| 51 | Pdk4 | -1.50 | -7.89 | 2.51 | 4.93E-05 | 0.025 | Down |
| 52 | Plin5 | -1.55 | -7.71 | 2.35 | 5.86E-05 | 0.027 | Down |
| 53 | Car4 | -1.62 | -7.42 | 2.11 | 7.67E-05 | 0.028 | Down |
| 54 | Lgals4 | -1.63 | -7.45 | 2.13 | 7.46E-05 | 0.028 | Down |
| 55 | Gm19277 | -1.65 | -9.33 | 3.57 | 1.46E-05 | 0.017 | Down |
| 56 | Tfrc | -1.68 | -11.05 | 4.59 | 4.16E-06 | 0.009 | Down |
| 57 | Acot2 | -1.79 | -15.77 | 6.48 | 2.75E-07 | 0.001 | Down |
| 58 | Col28a1 | -1.80 | -8.88 | 3.26 | 2.11E-05 | 0.017 | Down |
| 59 | 9430076G02Rik | -2.00 | -6.43 | 1.18 | 2.08E-04 | 0.045 | Down |
| 60 | Dnahc6 | -2.06 | -6.25 | 1.00 | 2.51E-04 | 0.048 | Down |
| 61 | Angptl4 | -2.10 | -9.03 | 3.36 | 1.86E-05 | 0.017 | Down |
| 62 | Tmem82 | -2.15 | -7.27 | 1.98 | 8.80E-05 | 0.031 | Down |
| 63 | Adamts16 | -2.19 | -6.59 | 1.34 | 1.75E-04 | 0.044 | Down |
| 64 | Adamdec1 | -2.21 | -6.04 | 0.78 | 3.15E-04 | 0.048 | Down |
| 65 | Gm6445 | -2.21 | -7.15 | 1.87 | 9.92E-05 | 0.032 | Down |
| 66 | Acot1 | -2.24 | -17.82 | 7.02 | 1.06E-07 | 0.001 | Down |
| 67 | Cyp1a1 | -2.45 | -6.96 | 1.70 | 1.19E-04 | 0.036 | Down |
| 68 | Acot3 | -2.53 | -6.37 | 1.13 | 2.20E-04 | 0.045 | Down |
| 69 | Scd4 | -2.70 | -13.05 | 5.53 | 1.18E-06 | 0.003 | Down |
| 70 | Foxa1 | -2.79 | -6.49 | 1.25 | 1.93E-04 | 0.044 | Down |
| 71 | Klkb1 | -2.86 | -8.28 | 2.82 | 3.50E-05 | 0.021 | Down |
| 72 | Slc25a34 | -3.39 | -13.30 | 5.63 | 1.02E-06 | 0.003 | Down |
| 73 | Fbp2 | -4.35 | -6.58 | 1.34 | 1.76E-04 | 0.044 | Down |
| 74 | Hmgcs2 | -5.34 | -10.30 | 4.18 | 7.03E-06 | 0.012 | Down |
| 75 | Ucp3 | -5.49 | -20.21 | 7.51 | 3.98E-08 | 0.001 | Down |

**Table S3.** Signaling Pathway Enrichment Analysis for Dysregulated Genes in Trophoblast Exposed to Silica Nanoparticles

| GOID | GOTerm | P Value | FDR | Genes (%) | Associated Genes Found |
| --- | --- | --- | --- | --- | --- |
| 00030 | Pentose phosphate pathway | 1.983E-03 | 3.967E-03 | 9.38 | Fbp2, Pfkl, Rgn |
| 00270 | Cysteine and methionine metabolism | 7.085E-03 | 7.085E-03 | 6.00 | Bcat1, Cbs, Cth |
| 03320 | PPAR signaling pathway | 2.924E-07 | 1.462E-06 | 9.41 | Angptl4, Cpt1a, Fabp5, Hmgcs2, Plin5, Scd1, Scd4, Slc27a1 |
| 04152 | AMPK signaling pathway | 5.915E-06 | 2.366E-05 | 6.35 | Acacb, Cpt1a, Fbp2, Lipe, Mlycd, Pfkl, Scd1, Scd4 |
| 00062 | Fatty acid elongation | 1.082E-04 | 3.246E-04 | 12.50 | Acot1, Acot2, Acot3, Acot4 |
| 01040 | Biosynthesis of unsaturated fatty acids | 1.488E-07 | 8.927E-07 | 18.75 | Acot1, Acot2, Acot3, Acot4, Scd1, Scd4 |
